# Weight Loss after 12 Weeks of Exercise and/or Nutritional Guidance Is Not Obligatory for Induced Changes in Local Fat/Lean Mass Indexes in Adults with Excess of Adiposity

**DOI:** 10.3390/nu12082231

**Published:** 2020-07-26

**Authors:** Robinson Ramírez-Vélez, Mikel Izquierdo, Karem Castro-Astudillo, Carolina Medrano-Mena, Angela Liliana Monroy-Díaz, Rocío del Pilar Castellanos-Vega, Héctor Reynaldo Triana-Reina, María Correa-Rodríguez

**Affiliations:** 1 Complejo Hospitalario de Navarra (CHN), Universidad Pública de Navarra (UPNA), Instituto de Investigación Sanitaria de Navarra (IdiSNA), 31008 Pamplona, Navarra, Spain; mikel.izquierdo@gmail.com; 2 Centro de Investigación Biomédica en Red de Fragilidad y Envejecimiento Saludable (CIBERFES), Instituto de Salud Carlos III, 28029 Madrid, Spain; 3 Centro de Acondicionamiento Físico y Nutrición, KCFIT, Santiago de Cali, Valle 760011, Colombia; gerencia@karemcastro.com; 4 Facultad de Organización Deportiva, Universidad Autónoma de Nuevo León, Nuevo León 66455, Mexico; medrano11.sc@gmail.com; 5 Programa de Bacteriología y Laboratorio Clínico, Facultad de Ciencias de la Salud-Universidad de Boyacá, Tunja 150003, Boyacá, Colombia; almonroy@uniboyaca.edu.co; 6 Grupo CORPS, Programa de Fisioterapia, Facultad de Ciencias de la Salud, Universidad de Boyacá, Tunja 150003, Boyacá, Colombia; dpcastellanos@uniboyaca.edu.co; 7 Grupo GICAEDS, Programa de Cultura Física, Deporte y Recreación, Universidad Santo Tomás, Bogotá 110311, Colombia; hectortriana@usantotomas.edu.co; 8 Department of Nursing, University of Granada, 18016 Granada, Spain; macoro@ugr.es

**Keywords:** interval training, strength training, body composition, fat mass, muscle mass, obesity, latinos

## Abstract

The objectives of this secondary analysis are (1) to investigate the differential effects of exercise training modalities–high-intensity interval training (HIIT), resistance training (RT), combined training (CT = HIIT + RT), and/or nutritional guidance (NG) alone–on local fat/lean mass indexes in adults with excess of adiposity; (2) to identify the individual patterns of response based on either a clinical criterion of weight loss (≥5%) and/or technical error (TE) of measurement of local fat/lean mass indexes; and (3) to assess the individual change for body composition parameters assigned either to HIIT, RT, CT, and/or NG groups utilizing a TE. A 12-week trial was conducted in 55 participants randomized to one of the four interventions. The primary outcome was clinical change in body weight (i.e., weight loss of ≥5%). Secondary outcomes included change in ratio of android and gynoid fat mass, as well as local fat and lean mass indexes (arms, trunk, and legs), before and after intervention. The main findings from the current analysis revealed that (i) after 12 weeks of follow-up, significant decreases in several body composition indexes were found including body weight, arm, trunk, and legs fat mass, and android and gynecoid fat mass were observed in HIIT, RT, and CT groups (*p* < 0.05); (ii) a significant proportion of individuals showed a positive response following 12 weeks of training, led by the HIIT group with 44% and followed by RT with 39% in 9 indexes; (iii) the HIIT group showed lowest rates of adverse responders with (6%); and (iv) the individual patterns of response utilizing clinically meaningful weight loss were not necessarily associated with the corresponding individual training-induced changes in body composition indexes in adults with excess of adiposity. Overall, the study suggests that HIIT has an important ability to reduce the prevalence of non-response to improve body composition indexes.

## 1. Introduction

Data from the Global Burden of Disease Study shows a steady increase in the prevalence of excess weight, and it has been projected that in 2030 there will be 2.16 billion overweight people in the world [1]. One-third of the population in Latin America is overweight or obese [2], and over half (56.5%) of the Colombian adult population (18 to 64 years old) are overweight+obesity (52.8% for men and 59.6% for women) [3]. The most common causes of excess weight are high energy density food consumption and a decrease in physical activity levels [4], and the migration from rural to urban areas can also contribute to these lifestyle changes [5]. It is well recognized that being excess weight not only has a significant, adverse impact on disease risk, but also has important consequences for health (e.g., cardiovascular disease, hypertension, stroke, type 2 diabetes mellitus, hyperlipidemia, kidney disease, liver and gall bladder disease, osteoarthritis, and certain cancers) and psychosocial (e.g., bulimia, anxiety, depression, body dissatisfaction, and low body- and self-esteem) functioning, and is related to poor quality of life [6]. Accordingly, efforts to prevent, reduce, or intervene in weight gain and obesity are at the forefront of public health priorities [7].

Recent epidemiological studies indicate that the location of adipose tissue deposits (i.e., body fat distribution) is the main predisposing factor for the development of metabolic abnormalities and other obesity-related co-morbidities [8,9]. Moreover, as individuals gain weight, their body composition changes through the accruement of proportionately more fat than lean mass. In this line, Hu et al. [10] reported a higher risk for cardiometabolic disorders related to high levels of trunk adiposity and low levels of leg adiposity in white and African American adults. Similarly, Choi et al. [8] showed that higher leg fat mass was associated with a lower risk of type 2 diabetes mellitus in a Korean population. By contrast, other studies have found inverse associations between leg/trunk adiposity and blood pressure [11], subclinical atherosclerosis [12], dyslipidemia [10], and metabolic syndrome [13].

Due to the cost to both physical (i.e., metabolic and/or biomechanical disorders) [14,15] and psychological health (i.e., depression, dementia, and cognitive/skills process) [16,17], many clinical studies have been conducted on various interventions to improve body weight and composition [18,19]. A growing body of literature demonstrates that in comparison with dietary restriction alone, exercise, either accompanied by weight loss or not, can lead to favorable changes in body composition/function, including a reduction in metabolic abnormalities and abdominal adiposity, and improves the fat free mass to total mass ratio [20,21,22,23,24]. However, only one study to our knowledge [22] has tested whether nutritional guidance (NG) in conjunction with different exercise training modalities—including high-intensity interval training (HIIT), resistance training (RT), or combined training (CT = HIIT + RT)—might be more effective and provide additional improvements on body composition in overweight and obese adults.

Individual differences in inherited and acquired phenotypic characteristics may modify the response to a given exercise training modality, resulting in substantial interindividual variability. This means that, under the same stimulus, while some individuals may achieve benefits after training (responders), others can present an unchanged or worsened response (non-responders). However, in human trials, the veracity of the approach to determine the existence of individual variability has been questioned [25]. In adults, interindividual variability in health biomarker responses to exercise training, such as blood pressure, insulin resistance parameters, lipids profile, muscle strength, and cardiorespiratory fitness (CRF), have not been fully clarified [21,22,23,24,25,26,27,28,29,30], while there is scarce evidence in the physically inactive/overweight adults [26,27,28]. Recently, statistical approaches use cut-off points for identifying responders/non-responders considering both biological and TE measurement [29]. In doing so, they try to improve the confidence when classifying responders as non-responders and vice versa. The rationale for this surrogate approach is that sufficiently large individual changes are unlikely to be due simply to error of measurement and day-to-day variability and can therefore be considered significant changes [25]. The idea of responsiveness to an intervention does not only pertain to exercise physiology, as personalized medicine has recently gained momentum in the fields of pharmacology [30], nutrition [31], or exercise interventions [32,33].

The combination of physical inactivity and excess weight is highly relevant in Latin America, especially in Colombia, and is associated with noncommunicable diseases [2,3]. Due to the fact that Hispanics with obesity have higher mortality rates from cardiovascular disease, type 2 diabetes mellitus, cancer and lower rates of self-reported physical activity than white Americans [34], there is a need to understand these differences and their clinical implications.

Research to date has focused on the variability of CRF in response to exercise [25,26,29], whereas anthropometric measures including body weight, or local and overall body composition parameters have received less attention. To our knowledge, no study has examined individual variability for weight loss response to exercise in subjects with excess weight and few have investigated the response for change in body weight [35,36,37,38]. The objectives of this secondary analysis are (1) to investigate the differential effect of exercise training and/or NG on local lean mass/fat outcomes; (2) to assess the individual change for body composition indexes assigned to either HIIT, RT, CT, or NG groups utilizing a TE of measurement; and (3) to identify the individual patterns of response based on a clinical criterion (weight loss ≥ 5%), plus response based TE on local fat/lean mass indexes. Based on the benefits previously reported in body composition markers with lifestyle intervention [39,40], we hypothesized that the magnitude of change in weight loss (≥5%) after 12 weeks of intervention would not be associated with magnitude of change in local body composition parameters in sedentary adults with excess of weight.

## 2. Materials and Methods

### 2.1. Design and Study Population

The original trial is registered at ClinicalTrials.gov (NCT02715063) [41]. Full details of the original trial protocol are published [41,42]. The present study was conducted from March 2016 to June 2017 in Bogotá, Colombia. The study received ethical approval from the Ethics Committee of The University of Manuela Beltran (ID 06-1006-2014) and complied with the revised ethical guidelines of the Declaration of Helsinki (revision of 2013). Briefly, the study included a total of 55 sedentary subjects (*n* = 23, 42% males), no participation in exercise more than once a week for the previous six months, aged 30–50 years, with excess of weight defined according to the with body mass index (BMI) ≥ 25 and ≤ 35 kg/m^2^ and/or with abdominal obesity: waist circumference (WC) at least 90 cm for men, and at least 80 cm for women were included in the study. Participants were recruited from a private healthcare institution (Clínica Rangel Pereira, Bogotá, Colombia) and the Rosario University in Bogotá. All participants provided written informed consent.

**Figure 1:**
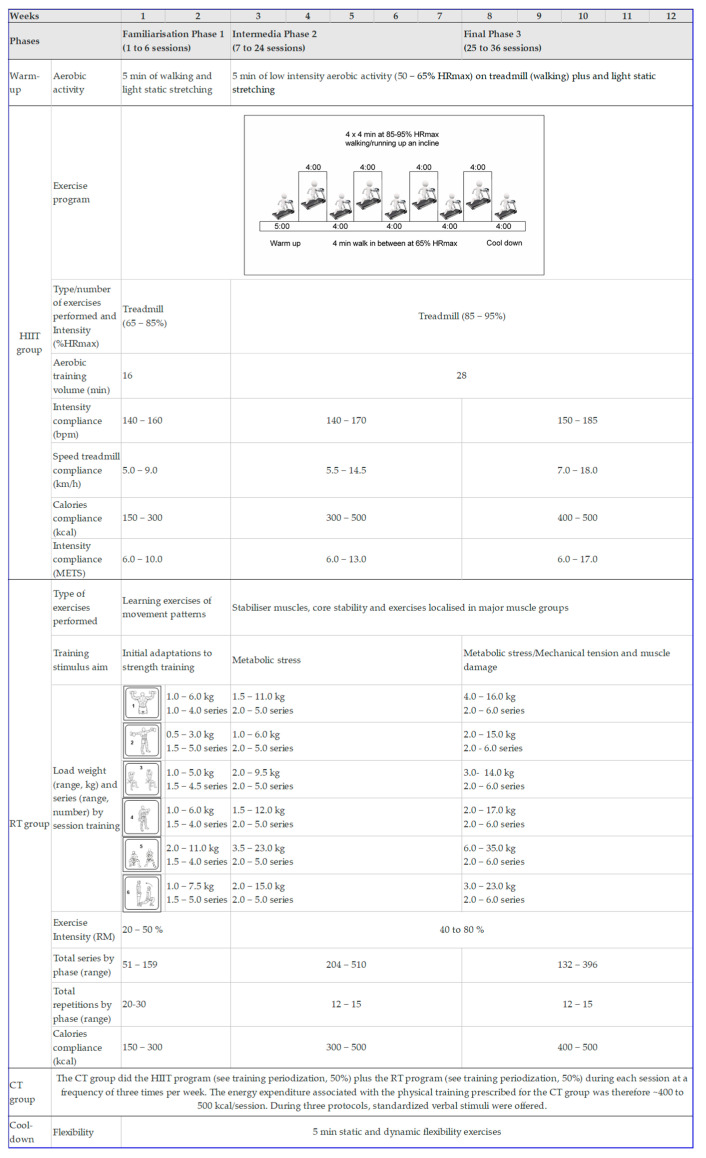
Training periodization of the study (ClinicalTrials.gov ID: NCT02715063). HIIT: high-intensity interval training group; RT: resistance training group; CT: combined training (HIIT + RT) group. HR: heart rate; RM: repetition maximum. Bpm: beats per min (heart rate). To RT group, external load was adjusted weekly to maintain the % of the 1RM (from 40% to 80% of 1RM) and total number of repetitions per exercise (12 to 15 repetitions). The intensity of the exercises increased individually and progressively according to the participants’ response on each day of exercise. The caloric cost of exercise session was calculated based on the one metabolic equivalent (MET) criteria, defined as the amount of oxygen consumed while sitting at rest with a value of 3.5 mL O_2_ per kg body weight × min. Additionally, assistance was provided to subjects during the exercise to complete the proposed RM.

In this extension study, novel experiments were also conducted to determine the TE of measurement for local fat mass/lean indexes and to assess whether changes in weight loss related to changes in local fat mass/lean indexes following NG, and/or different exercise training modalities—HIIT, RT, or CT (HIIT + RT) in excess weight adults (see statistical analysis section for more details). Details about interventions have been described [41]. To summarize, in order to compare the effects of NG (without exercise), and/or three exercise interventions (high-intensity interval training (HIIT), resistance training (RT), and a combined training (CT = HIIT + RT) protocol), all eligible participants were randomly assigned into 4 groups. A highly qualified physiotherapist and physical educator supervised each training session. The exercise program was individualized and included measurements of vital signs at the beginning, during, and the end of each session (rating of perceived exertion, heart rate, and energy expenditure). Permuted-block randomization was performed by a third-party to allocate all participants into the groups in a 1:1:1:1 ratio using a computer-generated random number sequence. Research staff/outcome assessors were blinded to the group status of the participants. Subjects were provided with customized dietary plans (percentages of total energy: carbohydrate, 45–65%; fat, 20–35%; and protein, 10–35%), designed by an experienced nutritionist. No vitamins or other nutritional complements were prescribed. NG participants did not practice any kind of supervised physical exercise/activities during the 12-week intervention. After interventions, baseline measurements (body composition) were performed, and post intervention measurements after 12 weeks [42]. A full description of the supervised exercise interventions is shown in Figure 1.

### 2.2. Procedures

Details of the interventions have been published elsewhere [42]. Briefly, the body weight (Tanita BC-418, Tokyo, Japan), height (Seca 274, Hamburg, Germany), and WC (Lufkin W606PM, Apex Tool Group, Lufkin, Mexico) were measured in duplicate using standard protocols. All measurements were assessed by trained dietitian specialists, and the same specialist performed each measurement. The TE of measurement values was less than 2% for all anthropometric variables. Dietary data were collected at baseline and post-intervention using 24-h records (one weekday and one weekend day). The Food Intake Analysis Software (FAO/INFOODS, Report of the Technical workshop on standards for food composition data interchange, Rome, Italy) and the guidelines in Colombia by the Colombian Institute of Family Welfare (in Spanish, Recomendaciones de Ingesta de Energía y Nutrientes-RIEN), were used to analyze total energy and macronutrient intake of each subject’s 24-h diet. Each participant met with the study dietician for nutrition assessment and counselling, and an individualized nutrition intervention plan was developed from the baseline food intake assessment according to the participant’s preferences. Periodic consultations were held on which the quality and quantity of meals were analyzed and, if necessary, minor adjustments were made.

In this extension study, the primary endpoint was change in body weight, based on weight loss of ≥5% in each participant. Secondary endpoints included change in local fat and lean mass indexes in arms, trunk, and legs, as well the ratio of android and gynoid fat mass, before and after intervention. All measurement scans were undertaken in a whole-body mode on a pencil beam densitometer scanner (Hologic QDR-1500 densitometer, GE Healthcare, Madison, WI, USA) with the analysis being performed using GE enCORE v.13.60 software (GE Healthcare, Madison, WI, USA), followed by manual correction of analysis markers when necessary to ensure appropriate identification of the arms, trunk, and legs. The trained personnel (MSc staff) acquired scans and analyzed everything in a routine research manner following standard operating procedures based on published recommendations [43].

Secondary endpoints for distribution of lean mass/fatness (i.e., grams or percentage) were calculated/determined at these sites in relationship to total body mass. Baseline characteristics are shown in Table 1.

### 2.3. Classification of Responders and Non-Responders

From the perspective of establishing a validated criteria for evaluating effectiveness of weight loss interventions, a 5% criterion appears to be well justified since it may bring benefits in some risk factors and for some patients [44]. Blackburn [45] in 1995 suggested that 5% might be a valid “single” criterion to assess significant weight loss, and data from the American Diabetes Prevention Program trial by Hamman et al. [46] showed that 5% weight loss would produce about 50% reduction in the incidence of type 2 diabetes. To quantify interindividual variability in response to each intervention and local fat mass/lean indexes, we calculated a TE measurement based on the methods outlined by Bouchard et al. [47] and as originally used in the National Health and Nutrition Examination Survey [48].

TE is calculated by taking the square root of the sum of squared differences of repeated measurements divided by the total number of paired samples multiplied by 2. Any local fat mass/lean indexes more than 2 × TE was considered a response for each secondary endpoint. The odds of an individual change that is greater than 2 × the TE being a true physiological change are 12:1 [49]. The cut-points were established as follows: arms fat mass (1.20 × 2 = 2.4%), trunk fat mass (0.71 × 2 = 1.44%), legs fat mass (0.68 × 2 = 1.36%), arms lean mass (102 × 2 = 204 g), trunk lean mass (373 × 2 = 746 g), legs lean mass (275 × 2 = 550), android fat mass (0.6 × 2 = 1.2%), gynecoid fat mass (1.0 × 2 = 2.0%), and android/gynecoid ratio (0.03 × 2 = 0.06%). Rate for response was calculated on the basis of the number of individuals who met more than 2 × TE calculation measurements per intervention group and 9-fat/lean mass parameters.

### 2.4. Statistical Analysis

All statistical analyses were performed using SPSS software, version 25.0 (IBM Corp., Armonk, NY, USA). The Shapiro–Wilk test was used to determine whether parametric tests were appropriate. A non-parametric test equivalent was applied if the assumption of normality was still rejected after log transformation of data, when necessary. To aid interpretation, data were back-transformed from the log scale for presentation in the results (i.e., arm muscle mass variable). All values are presented as mean, standard deviation (SD) or 95% confidence interval (95% CI) unless stated otherwise. The post-hoc/retrospective sample size was determined from Byrd et al. [21], assuming a power of 0.90 and an effect size of 0.8 in primary endpoint (body weight). Therefore, 14 subjects would be needed for each of the four groups (total *n* required = 56). A general linear model of repeated measures was used to determine changes in local fat/lean mass distribution over the 12 weeks of follow-up with the treatment group and time as factors. The difference in this model was established with the Greenhouse–Geisser test, also considering the partial-eta squared (η*p*^2^) value as a measure of effect size, and the Tukey’s HSD test was used for the post hoc analysis. Cohen’s effect size (Cohen *d*) was calculated, and considered between 0.20–0.49 as small, 0.50–0.79 as moderate, and ≥ 0.80 was considered as large. Furthermore, the McNemar test was applied to compare the proportion between responders and non-responders for each group. All results with *p*-values less than 0.05 were considered statistically significant.

## 3. Results

### 3.1. Change in Primary Endpoit

Table 2 lists the effects of the four interventions on anthropometric and fatness indexes. In per-protocol analyses, body weight did not change in the NG group −1.1 kg (95% CI = −3.0 to 0.7, *d* = 0.32). Weight decreased in all measurements groups, by −4.5 kg (95% CI = −7.0 to −1.9, *d* = 0.97) in the HIIT group (*p* < 0.01 vs. NG group), −4.8 kg (95% CI = −8.0 to −1.6, *d* = 0.94) in the RT group (*p* < 0.01 vs. NG group), and −1.7 kg (95% CI = −3.4 to 0.0, *d* = 0.57) in the CT group, but not the group factor differences between the NG, RT, or HIIT groups (*p* =  0.109; η*p*^2^ = 0.130).

### 3.2. Response Prevalences by Clinically Meaningful Weight Loss (Primary Endpoit)

**Figure 2:**
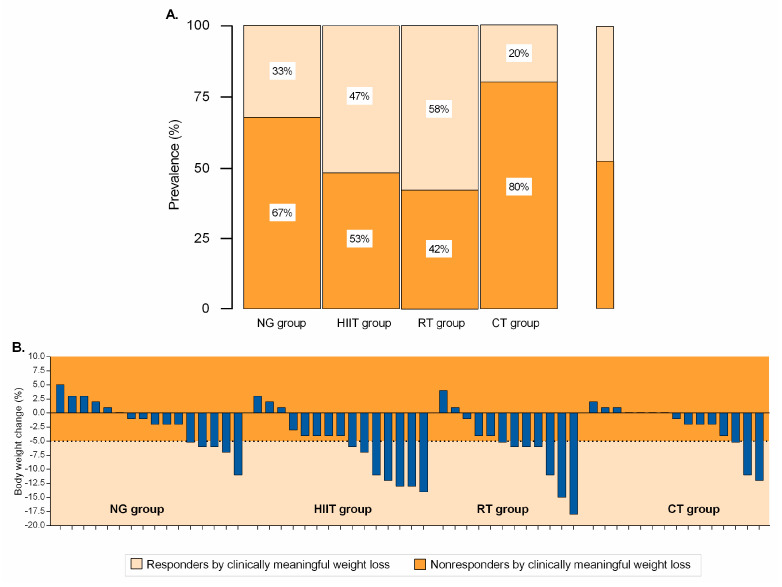
Categorical (Panel **A**) and data of all individual subjects (Panel **B**) for the intervention group at week 12, based on weight loss of ≥5%. Responders by clinically meaningful weight loss is illustrated by the lighter shaded area. Values within the darker shaded area represent nonresponse. NG: nutritional guidance alone group; HIIT: high-intensity interval training group; RT: resistance training group; CT: combined training (HIIT + RT) group.

Significant heterogeneity was apparent in the participants reaching ≥5% weight loss with responder rates of 33% (*n* = 5) for NG, 47% (*n* = 7) for HIIT, 58% (*n =* 7) for RT, and 20% (*n* = 3) for CT (all *p* < 0.001), Figure 2A,B.

### 3.3. Change in Secondary Endpoints

In regard to local fatness parameters, arms fat mass decreased in all intervention groups, by −2.1% (95% CI = −3.2 to −0.9, *d* = 0.99) in the HIIT group, −2.6% (95% CI = −4.2 to −1.1, *d* = 1.02) in the RT group, −1.8% (95% CI = −3.2 to −0.4, *d* = 0.75) in the CT group, and −1.2% (95% CI = −2.2 to −0.2, *d* = 0.65) in the NG group (*p* < 0.05), but not the time × group interaction (*p* =  0.097; η*p*^2^ = 0.114). Trunk fat mass changes in −3.4% (95% CI = −5.0 to −1.8, *d* = 1.22) in the HIIT group (*p* < 0.001 vs. NG group), −4.0% (95% CI = −6.5 to −1.4, *d* = 0.99) in the RT group (*p* < 0.01 vs. NG group), and −1.8% (95% CI = −3.1 to −0.5, *d* = 0.47) in the CT group. Significant decrease was observed for the HIIT group vs. the NG group −2.3% (95% CI = −4.4 to −0.1), *p* < 0.05 and the RT group vs. the NG group 2.9% (95% CI = −5.6 to −0.1), *p* < 0.05; time × group interaction (*p* = 0.049; η*p*^2^ = 0.149). Legs fat mass decreased in the HIIT group −2.4% (95% CI = −3.2 to −1.5, *d* = 1.64), RT group −2.1% (95% CI = −3.9 to −0.3, *d* = 0.72), and in the NG group −1.3% (95% CI = −2.2 to −0.5, *d* = 0.86), but not the time x group interaction (*p* = 0.230; η*p*^2^ = 0.080), Table 2.

When comparing within-group changes, the HIIT group, RT group, and CT group demonstrated a decrease for android fat mass (%), and gynoid fat mass (%) at week 12 compared with baseline (range = 1.1% to 4.1%); however, the training response (mean changes) difference between the four groups was not statistically significant (*p* = 0.197; η*p*^2^ = 0.087). There were no significant intervention effects with regard to lean mass indexes (within-group change from baseline to 12 weeks or intergroup difference in change from baseline to 12 weeks), Table 2.

### 3.4. Differences by Mode of Intervention in the Proportion of Responders

**Figure 3:**
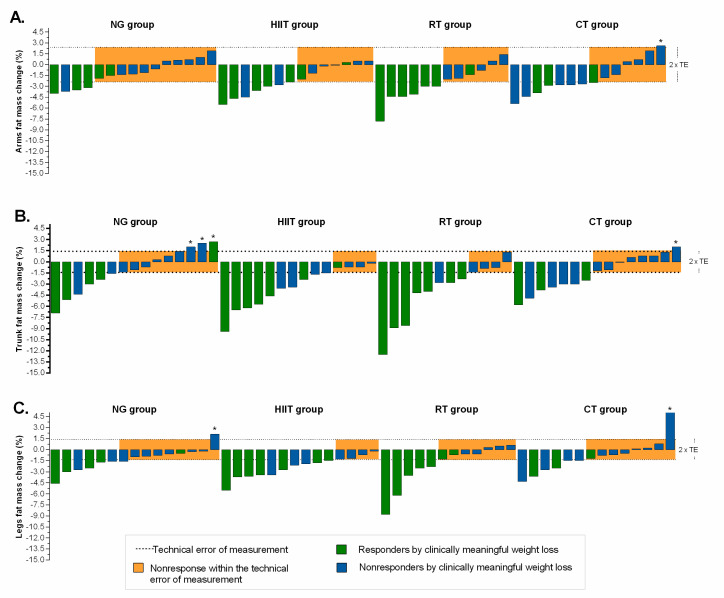
Individual change by intervention group based on fatness indexes. (Panel **A**): arms fat mass; (Panel **B**): trunk fat mass; and (Panel **C**): legs fat mass. The TE for each measurement (see “Methods” section for 2 × TE calculation) is illustrated by the lighter shaded area. Dashed lines represent the TE, while an individual falling within the shaded area would have demonstrated a nonresponse for both variables. Responders by clinically meaningful weight loss (≥5%) is illustrated by the green bar. Individual changes in blue bar represent nonresponse by clinically criterion weight loss (< 5%). *Adverse responders (individuals whose fat mass indexes increased by more than 2 × TE). NG: nutritional guidance alone group; HIIT: high-intensity interval training group; RT: resistance training group; CT: combined training (HIIT + RT) group.

**Figure 4:**
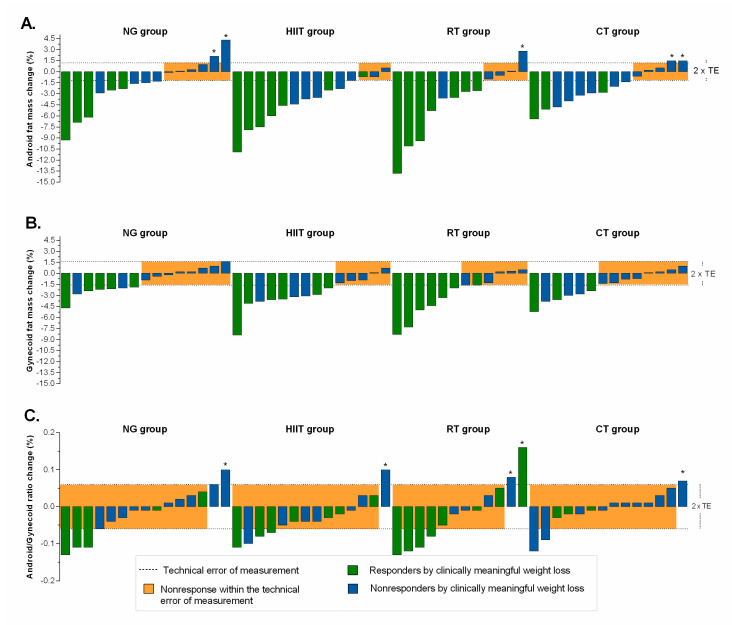
Individual change by intervention group based on distribution fatness indexes. (Panel **A**): android fat mass; (Panel **B**): gynecoid fat mass; and (Panel **C**): android/gynecoid ratio fat mass. The TE for each measurement (see “Methods” section for 2 × TE calculation) is illustrated by the lighter shaded area. Dashed lines represent the TE, while an individual falling within the shaded area would have demonstrated a nonresponse for both variables. Responders by clinically meaningful weight loss (≥5%) is illustrated by the green bar. Individual changes in blue bar represent nonresponse by clinically criterion weight loss (< 5%). *Adverse responders (individuals whose fat mass indexes increased by more than 2 × TE). NG: nutritional guidance alone group; HIIT: high-intensity interval training group; RT: resistance training group; CT: combined training (HIIT + RT) group.

The magnitude of individual responders for fatness indexes, relative to TE, plus clinically meaningful weight loss are presented in Figure 3 and Figure 4. Relatively moderate to high response rates were found (NG = 27%, HIIT = 50%, RT = 50%, and CT = 50%) for arms fat mass (Figure 3A); (NG = 40%, HIIT = 71%, RT = 67%, and CT = 50%) for trunk fat mass (Figure 3B); (NG = 40%, HIIT = 71%, RT = 42%, and CT = 43%) for legs fat mass (Figure 3C); (NG = 60%, HIIT = 79%, RT = 67%, and CT = 64%) for android fat mass (Figure 4A); (NG = 47%, HIIT = 64%, RT = 50%, and CT = 43%), for gynecoid fat mass (Figure 4B), and (NG = 13%, HIIT = 7%, RT = 17%, and CT = 7%), for android/gynecoid ratio, (Figure 4C). Adverse responders (individuals whose fatness indexes increased by more than 2 × TE) were observed (CT = 7%) for arms fat mass; (NG = 20%, and CT = 7%) for trunk fat mass; (NG = 7%, and CT = 7%) for legs fat mass (Figure 3, illustrated by asterisk); (NG = 13%, RT = 8%, and CT = 14%) for android fat mass; and (NG = 7%, HIIT = 7%, and RT = 17%), for android/gynecoid ratio, (Figure 4, illustrated by asterisk).

**Figure 5:**
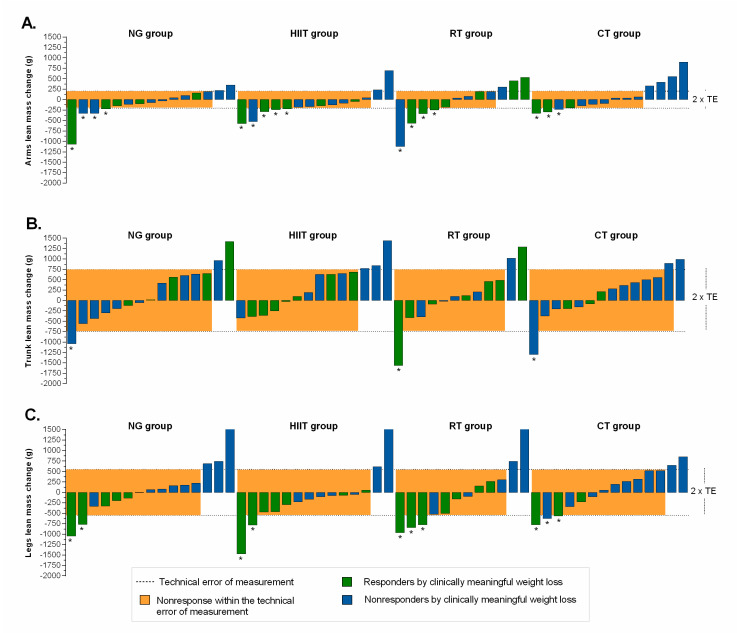
Individual change by intervention group based on lean mass indexes. (Panel **A**): arms lean mass; (Panel **B**): trunk lean mass; and (Panel **C**): legs lean mass. The TE for each measurement (see “Methods” section for 2 × TE calculation) is illustrated by the lighter shaded area. Dashed lines represent the TE, while an individual falling within the shaded area would have demonstrated a nonresponse for both variables. Responders by clinically meaningful weight loss (≥5%) is illustrated by the green bar. Individual changes in blue bar represent nonresponse by clinically criterion weight loss (< 5%). *Adverse responders (individuals whose lean mass indexes decreased by more than 2 × TE). NG: nutritional guidance alone group; HIIT: high-intensity interval training group; RT: resistance training group; CT: combined training (HIIT + RT) group.

Regarding region lean mass distribution, the proportion of responders by intervention groups were as follows (NG = 13%, HIIT = 14%, RT = 25%, and CT = 29%) for arms lean mass (Figure 5A); (NG = 13%, HIIT = 21%, RT = 17%, and CT = 7%) for trunk lean mass (Figure 5B); and (NG = 20%, HIIT = 14%, RT = 17%, and CT = 14%) for legs lean mass, (Figure 5C). Adverse responders (individuals whose lean mass indexes decreased by more than 2 × TE) were observed (NG = 27%, HIIT = 36%, RT = 33%, and CT = 21%) for arms lean mass; (NG = 7%, RT = 8%, and CT = 7%) for trunk lean mass; (NG = 13%, HIIT = 14%, RT = 25%, and CT = 21%) for legs lean mass, (Figure 5, illustrated by asterisk).

### 3.5. Total Prevalence of Variables Classified as Responders and Adverse Responders

**Figure 6:**
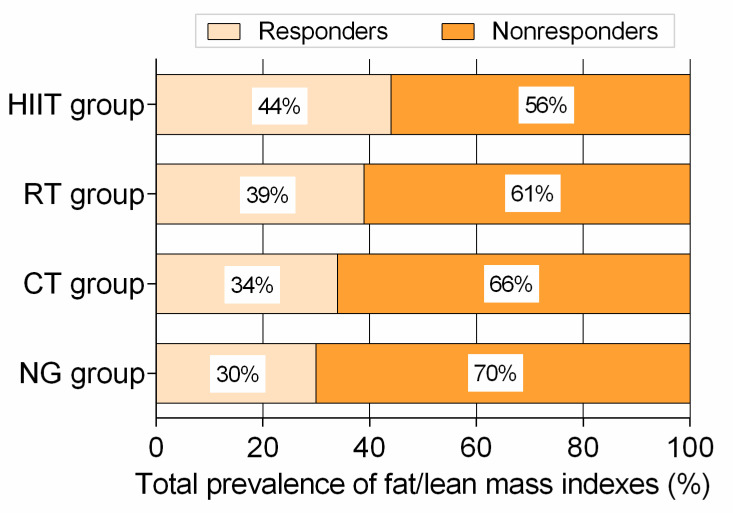
Total prevalence of responders vs. non-responders according to technical error of measurement for 3 fat mass, 3 lean mass, and 3 fatness distribution-related from each group intervention at week 12. NG: nutritional guidance alone group; HIIT: high-intensity interval training group; RT: resistance training group; CT: combined training (HIIT + RT) group.

Considering all 9 fat/lean mass endpoints, the HIIT group showed moderate rates of responders with 44%, followed by RT with 39%, CT with 34%, and NG with 30% all variables relative to 2 × TE (Figure 6). Similarly, the HIIT group showed lowest rates of adverse responders with 6%, followed by the RT, CT, and NG groups with 10% variables relative to 2 × TE (illustrated by asterisk in Figure 3 to Figure 5).

### 3.6. Adherence and Adverse Events

To qualify for adherence, participants needed to attend at least 27 of 36 prescribed exercise sessions (≥75% adherence) during the run-in period. From baseline to 12 weeks, the median exercise training adherence was 95% in the HIIT group; 96% in the RT group and 88% in the CT group, with no significant differences between groups (*p* = 0.671). No physical limitations or health problems were found during the training intervention (adverse events).

## 4. Discussion

Considering the fact that people exhibit a specificity of response, inducing a wide interindividual variability in the adaptations of exercise training, we aimed to verify the individual prevalence of responsiveness on different fat/lean mass indexes after 12 weeks of follow-up. The main findings from the current analysis revealed that (i) significant decreases in several body composition indexes including body weight, arms, trunk, and legs fat mass, and android and gynecoid fat mass were observed after HIIT, RT, and CT interventions; (ii) a significant proportion of individuals demonstrate a positive response following 12 weeks of HIIT intervention (44%), followed by RT (39%) in 9 fat/lean mass indexes; (iii) the HIIT group showed lowest rates of adverse responders with 6%; and (vi) the individual patterns of response following a clinically meaningful weight loss of ≥5% were not necessarily associated with the corresponding individual training-induced changes in body composition parameters in subjects with excess weight. These findings indicate that the prevalence of responders depends on the body composition outcome assessed.

To date, there is limited data regarding the non-responder prevalence for different training modalities such as HIIT, RT, and CT in sedentary and overweight/obese adults [26,35,50,51]. Our data reveal a wide interindividual variability for responders and non-responders in the magnitude of change in each body composition marker. Similarly, Alvarez et al. [27] found significant differences in the non-responder prevalence between the HIIT and RT groups for a decrease in fat mass, muscle mass, and tricipital skinfold in a cohort of sedentary insulin-resistant adult women. Along the same line, King et al. [35] found that there was a large inter-individual variability in weight change and compensatory responses after a 12-week exercise intervention in overweight and obese sedentary men and women. Gremeaux et al. [51] also reported a discrepancy at the individual level between body mass, trunk fat mass, and fat-free mass changes, suggesting a high interindividual variation of body composition in obese subjects after a 9-month lifestyle intervention with HIIT.

To elucidate the response prevalence after different training modalities such as HIIT, RT, and CT in sedentary and overweight/obese individuals might be valuable to establish efficient exercise programs. In our study cohort, HIIT elicited the lowest prevalence of non-responders considering all body composition outcomes. In accordance with our results, the HIIT program also resulted in few cases of non-responder subjects in trunk fat mass and found more effect in the decreasing of the tricipital, suprailiac, and abdominal skinfolds and fat mass [27]. Thus, the HIIT modality should be recommended to change body composition since it seems to be the most effective regimen to reduce the prevalence of non-responders considering body composition indexes versus other exercise training modalities.

Exercise training is an established cornerstone of any treatment plan for overweight or obese subjects irrespective of weight loss goals [52]. In this line, Willis et al. [53] reported that aerobic training is the optimal mode of exercise for reducing fat and body mass in middle-aged, overweight/obese individuals when compared with RT or a combination of aerobic training and RT. In line with our findings, they indicated that CT did not result in significantly more fat or body mass reductions over HIIT alone [53]. This contrasts with another study showing that a 12-week training program of combined exercise (moderate-interval training and RT) had greater benefits for weight loss and fat loss than HIIT or RT modalities in overweight and obese adults [23]. In the present study, the combination of HIIT and RT did not have an additive effect on improving body mass and body composition compared with HIIT or RT alone, suggesting that combination training regimens do not provide significant further benefit. Differences in the overall training dose (moderate training versus HIIT) in addition to the degree to which subjects were supervised likely accounts for the inconsistency in these findings. In the study by Ho et al. [23], the intervention involved five days per week of exercise, but the training was supervised only on three days per week, as the other two days could be completed at home.

We found no significant differences in lean mass parameters between baseline and after 12 weeks of intervention for any of the exercise training modalities. This contrasts with the findings of a 26-week study where obese older adults were assigned to a weight-management program plus one of three exercise programs (aerobic training, RT, or combined aerobic and RT) or to a control group (no weight-management or exercise program) [54]. The authors showed that lean mass decreased less in the combination and resistance groups than in the aerobic group. Considering that the aforementioned study used a long training period, it could be hypothesized that only long-term interventions might have substantial effect on lean mass. Nevertheless, since this is the first study to examine efficacy of HIIT, RT, CT, and NG on lean mass parameters, more studies are warranted in similar cohorts.

Independently of weight loss, the HIIT group showed the greatest improvements in fat mass indexes and the lower prevalence of non-response among the four trials, pointing to this training regimen as an effective means of improving fat distribution. Similarly, it has been previously [55,56,57] shown that HIIT induces clinically significant changes in body composition in adults with excess of adiposity [56], such as decreases in whole-body fat mass and waist circumference [57]. For example, Keating et al. [55] found that both HIIT and continuous exercise training reduced gynoid fat relative to baseline values, whereas no significant reduction in android fat was observed after 12 weeks of HIIT in inactive overweight adults, thereby suggesting that changes in adiposity could be dependent on exercise intensity. Nevertheless, these authors did not report the individual prevalence of responsiveness among individuals. In regard to interindividual variability, Álvarez et al. [27] found a higher prevalence of non-responders for body fatness following a HIIT (17%), or a RT (18.5%), respectively, while Gremeaux et al. [51] reported that 7.2% of participants were non-responders for a decrease in waist circumference. It is plausible that regional and whole-body fat reduction may occur differently between HIIT, RT, and/or CT exercise regimes, primarily because of mechanistic factors related to mitochondrial adaptations, change in energy expenditure, or excess post-exercise oxygen consumption [57]. However, it is not known which mode of training may induce an increased or decreased number of responders after interventions, and the causes of this phenomenon are still unknown.

The lower response in several endpoints in our CT subjects was unexpected, and clashes with the majority of the concurrent-training literature [58,59]. Not unlike our findings with respect to weight loss, the CT subjects in our study showed body composition adaptations that were comparatively similar to those found in the NG or RT group. These findings do support the existence of a plausible “interference phenomenon” between concurrent HIIT and RT with respect to body composition adaptations. In this line, some studies with adults, using different types of exercise, reported either a decrease or no change in body weight, accompanied by a decrease in body fat with concurrent training programs [60,61,62]. These findings have important implications for professionals designing exercise programs to improve body composition in overweight/obese and sedentary adults.

The main limitation, which is common to most reports in this field, is related to the vast variety of definitions of the response to an intervention in order to discern the systematic change and the interindividual variability from the intra-individual or the random variability. Secondly, genetic factors or energy metabolism that could be determinants of the interindividual variability were not measured in the present study. Thirdly, it should be noted that diets in the all groups were monitored by means of 24 h dietary recall. Although 24 h diet recall is recognized as a reliable method to collect a variety of detailed information about food consumed over a specific period, the tool has inherent limitations [63]. Fourthly, threshold-based dichotomous classification could overestimate the prevalence of non-responders [64]. It may be speculated that the heterogeneous prevalence of responders for each body composition parameter could be explained by the different cut-point used for the definition of responders (i.e., 2 × TE calculation); however, dichotomously classification based 2 × TE is a relatively robust threshold for the classification of “responders” [49]. Furthermore, the training in this study was performed under supervised condition, and this could limit the generalizability of the findings to a non-supervised group. Despite these limitations, this study is the first to our knowledge to assess the effect of different exercise training modalities and/or NG on body composition markers in Latin American adults and provides individual training-induced changes and NR differences between different training modalities. Moreover, body composition parameters were assessed by dual-energy X-ray absorptiometry, considered the “gold standard” for body composition measurements. Other strengths of our research include its randomized controlled trial design and the high rate of adherence to the trial interventions.

## 5. Conclusions

In summary, 12 weeks of HIIT, RT, and CT programs decreased several adiposity markers in adults with excess of adiposity, but weight loss of ≥5% is not obligatory for induced changes in individual body composition parameters. HIIT elicited the lowest prevalence of non-responders considering all body composition indexes, supporting that it is the most effective regimen and should be promoted by clinicians as a time-efficient strategy that confers the best benefits to body composition in overweight/obese and sedentary adults. While we reported the positive effects of HIIT in body fat distribution indexes, the mechanism involved remains unclear and further research is warranted.

## Figures and Tables

**Table 1:** Baseline characteristics of study participants.

Characteristics	NG (*n*= 15)	HIIT (*n*= 14)	RT (*n*= 12)	CT (*n*= 14)	*p* Value
Anthropometric parameters					
Age, years	41.2 (7.6)	43.6 (7.2)	38.7 (6.0)	39.2 (6.8)	0.237
Body mass, kg	82.4 (16.4)	75.1 (10.8)	84.2 (11.5)	77.2 (23.1)	0.169
Body mass index, kg/m^2^	29.3 (3.9)	29.7 (2.7)	31.3 (3.7)	30.2 (3.8)	0.531
Waist circumference, cm	95.1 (12.4)	90.0 (8.6)	94.7 (8.3)	91.2 (7.3)	0.367
Educational level, *n* (%)^a^					
High school	2 (13)	1 (13)	2 (17)	1 (7)	0.188
Technician	8 (53)	5 (36)	0 (0)	4 (27)	
University	5 (33)	8 (57)	10 (83)	9 (64)	
Level of occupation, *n* (%)^a^					
Full timer	7 (47)	7 (44)	8 (50)	10 (71)	0.495
Half timer	1 (7)	2 (13)	0 (0)	1 (7)	
Independent	5 (33)	5 (31)	2 (13)	3 (21)	
Housewife	1 (7)	0 (0)	0 (0)	0 (0)	
Unemployed	1 (7)	0 (0)	2 (13)	0 (0)	
Socioeconomic status, *n* (%)^a^					
Low	1 (7)	1 (7)	2 (17)	1 (7)	0.651
Mid	14 (93)	13 (93)	8 (67)	11 (79)	
High	0 (0)	0 (0)	2 (17)	2 (14)	
Caloric distribution by nutrients					
Daily caloric intake, mean (SD)	1441 (471)	1595 (279)	1791 (439)	1811 (439)	0.060
Protein, %	19.8 (5.1)	17.4 (5.5)	18.2 (4.2)	18.4 (3.1)	0.221
Fat, %	37.7 (7.7)	35.4 (3.6)	38.1 (5.5)	35.2 (5.6)	0.435
Carbohydrate, %	42.7 (8.0)	47.3 (8.4)	43.7 (5.6)	46.8 (8.4)	0.195

Continuous variables are reported as mean values (standard deviations (SD) and categorical variables are reported as numbers and (%)^a^. Body mass index was calculated with the following formula = body weight (kg)/height squared (m^2^). To compare groups, ANOVA was applied from quantitative variables, while for the qualitative variables, the Chi-square test was used.

**Table 2:** Anthropometrics, body composition (fatness/lean mass) and distribution indices at baseline, and changes after 12 weeks by groups.

Variable	Baseline		12 Weeks		Within-Group Change from			Intergroup Difference in Change		
					Baseline to 12 Weeks			from Baseline to 12 Weeks		
	Mean (standard deviation)				Mean (95% Confidence Interval)					
Primary endpoint										
Weight (kg)										
HIIT group	75.1	10.8	70.6	11.2	−4.5	−7.0	−1.9 *		N.A	
RT group	84.2	11.5	79.4	13.2	−4.8	−8.0	−1.6 **		N.A	
CT group	77.2	23.1	75.6	22.7	−1.7	−3.4	0.0 *		N.A	
NG group	82.4	16.4	81.3	18.6	−1.1	−3.0	0.7		N.A	
HIIT group vs. NG group	N.A		N.A		N.A			−3.3	−6.4	−0.3 ^†^
RT group vs. NG group	N.A		N.A		N.A			−3.6	−7.0	−0.3 ^†^
CT group vs. NG group	N.A		N.A		N.A			0.6	−3.0	−1.8
CT group vs. HIIT group	N.A		N.A		N.A			2.8	−1.0	5.6
CT group vs. RT group	N.A		N.A		N.A			3.1	−0.1	6.3
Secondary endpoints										
Arms fat mass (%)										
HIIT group	40.2	7.4	38.3	7.3	−2.1	−3.2	−0.9 **		N.A	
RT group	37.8	10.4	35.1	9.8	−2.6	−4.2	−1.1 **		N.A	
CT group	40.4	8.6	38.6	8.7	−1.8	−3.2	−0.4 *		N.A	
NG group	33	6.4	31.8	7.3	−1.2	−2.2	−0.2 *		N.A	
HIIT group vs. NG group	N.A		N.A		N.A			−0.9	−2.3	0.6
RT group vs. NG group	N.A		N.A		N.A			−1.4	−3.1	0.3
CT group vs. NG group	N.A		N.A		N.A			−0.6	−3.1	1.9
CT group vs. HIIT group	N.A		N.A		N.A			0.4	−1.6	2.3
CT group vs. RT group	N.A		N.A		N.A			0.9	−1.3	3.1
Trunk fat mass (%)										
HIIT group	42.7	5.4	39.4	6.3	−3.4	−5.0	−1.8 ***		N.A	
RT group	43.1	5.6	39.1	7.3	−4.0	−6.5	−1.4 **		N.A	
CT group	44.3	6.9	42.1	7.7	−1.8	−3.1	−0.5 *		N.A	
NG group	41.7	3.8	40	5.4	-1.3	−2.8	0.3		N.A	
HIIT group vs. NG group	N.A		N.A		N.A			−2.3	−4.4	−0.1 ^†^
RT group vs. NG group	N.A		N.A		N.A			−2.9	−5.6	−0.1 ^†^
CT group vs. NG group	N.A		N.A		N.A			−0.7	−2.7	1.3
CT group vs. HIIT group	N.A		N.A		N.A			1.6	−0.4	3.6
CT group vs. RT group	N.A		N.A		N.A			2.2	0.4	4.8
Legs fat mass (%)										
HIIT group	37.5	7.4	35	7.2	−2.4	−3.2	−1.5 ***			
RT group	35.8	10.7	33.7	10.7	−2.1	−3.9	−0.3 *			
CT group	36.4	10	35.6	9.5	−0.7	−2.4	0.9			
NG group	30.8	7.3	29.6	7.5	−1.3	−2.2	−0.5 **			
HIIT group vs. NG group	N.A		N.A		N.A			−1.0	2.2	0.2
RT group vs. NG group	N.A		N.A		N.A			−0.8	−2.5	1.0
CT group vs. NG group	N.A		N.A		N.A			0.6	−1.2	2.3
CT group vs. HIIT group	N.A		N.A		N.A			1.6	−0.2	3.4
CT group vs. RT group	N.A		N.A		N.A			1.4	−1.0	3.7
Arms lean mass (g)										
HIIT group	4734.9	1131.8	4593.1	1179.4	−115.8	−295.4	63.8		N.A	
RT group	5608.7	1616.8	5553.3	1330.7	−55.4	−353.1	242.3		N.A	
CT group	4608.5	863.0	4707.2	1013.7	66.8	−139.9	273.5		N.A	
NG group	5976.6	1740.1	5753.3	1652.2	−88.9	−275.1	97.2		N.A	
HIIT group vs. NG group	N.A		N.A		N.A			−26.9	−274.1	220.4
RT group vs. NG group	N.A		N.A		N.A			33.5	−285.4	352.4
CT group vs. NG group	N.A		N.A		N.A			155.7	−108.7	420.2
CT group vs. HIIT group	N.A		N.A		N.A			182.6	−77.9	442.1
CT group vs. RT group	N.A		N.A		N.A			122.2	−212.6	457.0
Trunk lean mass (g)										
HIIT group	20,205.40	2990.6	20,619.90	2952.6	321.7	−5.5	648.9		N.A	
RT group	22,571.50	4144.4	22,672.60	4007.6	101.1	−362.8	565.0		N.A	
CT group	20,583.00	2221.1	20,759.10	2327.6	137.7	−200.0	475.4		N.A	
NG group	23,380.70	4727.9	23,141.60	4725.9	171.7	−187.4	530.7		N.A	
HIIT group vs. NG group	N.A		N.A		N.A			150.0	−315.4	615.5
RT group vs. NG group	N.A		N.A		N.A			−70.6	−617.4	476.2
CT group vs. NG group	N.A		N.A		N.A			−34.0	−505.6	437.7
CT group vs. HIIT group	N.A		N.A		N.A			−184.0	−631.4	263.4
CT group vs. RT group	N.A		N.A		N.A			36.5	−495.5	568.8
Legs lean mass (g)										
HIIT group	14,589.40	2723.2	14,452.40	2859.7	−129.0	−534.9	276.9		N.A	
RT group	17,128.30	3123.8	17,065.80	3441.3	−62.4	−542.4	417.6		N.A	
CT group	15,427.20	2717.7	15,578.90	3088.9	51.7	−241.3	344.7		N.A	
NG group	17,268.90	3936.2	16,995.20	3993.5	75.4	−292.0	442.8		N.A	
HIIT group vs. NG group	N.A		N.A		N.A			−204.4	−725.0	316.2
RT group vs. NG group	N.A		N.A		N.A			−137.8	−700.5	424.9
CT group vs. NG group	N.A		N.A		N.A			−23.7	−476.2	428.8
CT group vs. HIIT group	N.A		N.A		N.A			180.7	−295.6	657.0
CT group vs. RT group	N.A		N.A		N.A			114.1	−400.8	628.3
Distribution indices										
Android fat mass (%)										
HIIT group	44.9	6.9	41.0	7.7	−4.0	−5.8	−2.1 ***		N.A	
RT group	46.2	5.1	42.0	7.4	−4.1	−7.2	−1.1 **		N.A	
CT group	46.9	7.3	44.4	8.6	−2.1	−3.6	−0.7 **		N.A	
NG group	44.6	4.1	42.2	6.2	−1.8	−3.7	0.2		N.A	
HIIT group vs. NG group	N.A		N.A		N.A			−2.2	−4.8	0.4
RT group vs. NG group	N.A		N.A		N.A			−2.3	−5.6	1.0
CT group vs. NG group	N.A		N.A		N.A			0.3	−2.7	2.0
CT group vs. HIIT group	N.A		N.A		N.A			−1.9	−0.4	4.1
CT group vs. RT group	N.A		N.A		N.A			2.0	−1.0	5.1
Gynecoid fat mass (%)										
HIIT group	40.0	6.4	37.2	6.6	−2.6	−3.9	−1.3 ***		N.A	
RT group	38.9	10.8	36.1	11.1	−2.8	−4.7	−1.0 **		N.A	
CT group	40.7	9.1	38.9	9.2	−1.7	−2.7	−0.6 **		N.A	
NG group	35.0	7.1	33.9	7.7	−1.1	−2.0	−0.2 *		N.A	
HIIT group vs. NG group	N.A		N.A		N.A			−1.6	3.1	0.0
RT group vs. NG group	N.A		N.A		N.A			−1.7	−3.6	0.1
CT group vs. NG group	N.A		N.A		N.A			−0.6	−1.9	0.8
CT group vs. HIIT group	N.A		N.A		N.A			1.0	−0.6	2.6
CT group vs. RT group	N.A		N.A		N.A			1.2	−0.8	3.1
Android/Gynecoid ratio (%)										
HIIT group	1.1	0.2	1.1	0.2	0	−0.1	0		N.A	
RT group	1.3	0.3	1.2	0.4	0	−0.1	0		N.A	
CT group	1.2	0.2	1.2	0.2	0	0	0		N.A	
NG group	1.3	0.3	1.3	0.3	0	−0.1	0		N.A	
HIIT group vs. NG group	N.A		N.A		N.A			0	−0.1	0
RT group vs. NG group	N.A		N.A		N.A			0	−0.1	0
CT group vs. NG group	N.A		N.A		N.A			0	0	0.1
CT group vs. HIIT group	N.A		N.A		N.A			0	0	0.1
CT group vs. RT group	N.A		N.A		N.A			0	0	0.1

N.A: not applicable; NG: nutritional guidance alone group; HIIT: high-intensity interval training group; RT: resistance training group; CT = combined training (HIIT + RT) group; within-group change: * *p* < 0.05, ** *p* < 0.01, *** *p* < 0.001; between-group difference in change: ^†^ *p* < 0.05.
